# Superior Outcomes With Ommaya Reservoir in Sustained Intracranial Hypertension Control

**DOI:** 10.31083/RN44338

**Published:** 2025-12-25

**Authors:** Yuying Cen, Yuheng Shan, Xiaojiao Xu, Jiahua Zhao, Jiatang Zhang

**Affiliations:** ^1^Center of Neurology, Beijing Anzhen Hospital, Capital Medical University, 100029 Beijing, China; ^2^Department of Neurology, Characteristic Medical Center of Chinese People’s Armed Police Force, 300162 Tianjin, China; ^3^School of Medicine, Nankai University, 300191 Tianjin, China; ^4^Department of Neurology, The First Medical Centre, Chinese People's Liberation Army (PLA) General Hospital, 100853 Beijing, China

**Keywords:** intracranial hypertension, Ommaya reservoir, external ventricular drain, lumbar drainage, acute kidney injury, hipertensión intracraneal, reservorio de Ommaya, drenaje ventricular externo, drenaje lumbar, lesión renal aguda

## Abstract

**Background:**

Persistent intracranial hypertension (ICH) is a difficulty that must frequently be faced in the neuro- intensive care unit (ICU). The management of ICH is quite varied, and the choice of measures is determined by the experience of attending doctors. We aimed to evaluate the efficacy of different intervention measures in treating non-traumatic persistent ICH.

**Methods:**

A total of 119 non-traumatic intracranial hypertension cases treated in the neuro-ICU of the PLA General Hospital between 2010 and 2023 were retrospectively reviewed. Patients were divided into five groups according to the methods for controlling intracranial pressure (ICP). Based on the records of ICP, biochemical indicators, general status, and prognosis of patients in each group, the differences between groups and the differences within groups before and after intervention were compared. Repeated measures data of multiple groups were analyzed using generalized estimating equation (GEE) methods.

**Results:**

External ventricular drain (EVD), lumbar drainage (LD) and Ommaya reservoir (OR) had advantages in reducing ICP compared with the drug therapy alone (DT) group. Among them, the Ommaya reservoir exhibited optimal efficacy. Intervention with repeated lumbar puncture (LP) and the Ommaya reservoir effectively improved the general state of patients, evidenced by decreased mRS scores. The median creatinine value in the OR group decreased significantly at three months, suggesting that this method can moderate the renal burden. The OR group had the lowest probability of electrolyte imbalances and renal function damage, while the LD and EVD groups had a higher probability of pulmonary infection.

**Conclusions:**

The Ommaya reservoir is an effective and safe means of controlling ICP and thus has great potential in treating non-traumatic persistent ICH.

## Abstract (es)

**Antecedentes:**

La hipertensión intracraneal persistente (ICH, persistent intracranial hypertension) es una dificultad a la que hay que enfrentarse con frecuencia en la UCI neurológica. El tratamiento de la ICH es muy variado y la elección de las medidas dependerá de la experiencia de los médicos responsables. Nuestro objetivo fue evaluar la eficacia de diferentes medidas de intervención a la hora de tratar la ICH persistente no traumática.

**Métodos:**

Se revisaron retrospectivamente un total de 119 casos de hipertensión intracraneal no traumática tratados en la UCI neurológica del Hospital General del Ejército Popular de Liberación (PLA General Hospital) entre los años 2010 y 2023. Se dividió a los pacientes en cinco grupos, según los métodos utilizados para controlar la presión intracraneal (PIC). A partir de los registros de PIC, los indicadores bioquímicos, el estado general y el pronóstico de los pacientes de cada grupo, se compararon las diferencias entre los grupos y las diferencias dentro de los grupos antes y después de la intervención. Se analizaron los datos de medidas repetidas de múltiples grupos con métodos de ecuaciones de estimación generalizadas (EEG).

**Resultados:**

El drenaje ventricular externo (DVE), el drenaje lumbar (DL) y el reservorio de Ommaya (RO) presentaron ventajas en la reducción de la PIC en comparación con el grupo de tratamiento farmacológico único (TF). Entre ellos, el reservorio de Ommaya mostró una eficacia óptima. La intervención con punción lumbar (PL) repetida y el reservorio de Ommaya mejoraron eficazmente el estado general de los pacientes, tal y como demuestra la disminución de las puntuaciones mRS. El valor medio de creatinina en el grupo OR disminuyó significativamente a los tres meses, lo que sugiere que este método puede moderar la carga renal. El grupo del RO tuvo la menor probabilidad de desequilibrios electrolíticos y daño de la función renal, mientras que los grupos de DL y DVE tuvieron una mayor probabilidad de infección pulmonar.

**Conclusiones:**

El reservorio de Ommaya es un medio eficaz y seguro para controlar la PIC y, por lo tanto, tiene un gran potencial en el tratamiento de la ICH persistente no traumática.

## 1. Introduction

Persistent intracranial hypertension is a difficulty that must frequently be faced in the clinical work of the neuro- intensive care unit (ICU). Some common neurological disorders, such as intracranial malignant tumors, central nervous system (CNS) infections and cerebrovascular disease, can cause brain oedema and increased intracranial pressure (ICP) in the closed cranial cavity. Refractory intracranial hypertension (ICH) is usually defined as a condition where cerebral spinal fluid (CSF) opening pressure (by lumbar puncture) ≥250 mmH_2_O lasts for a period (about two weeks) under active medical treatment [1, 2, 3]. Prolonged and uncontrollable ICH can lead to convulsive seizures, consciousness disorders, as well as hearing and vision impairment [4, 5]. Cerebral herniation may occur and subsequently lead to death [6].

Conventional treatment options include pharmacological agents and repeated therapeutic lumbar puncture (LP) [5, 7, 8]. Mechanical drainage has gained popularity in recent years [9, 10]. In our study, we retrospectively analysed the clinical data of patients with refractory ICH caused by non-traumatic aetiologies who visited the Department of Neurology at the First Medical Center of the PLA General Hospital between January 2010 and June 2023. Efficacy analyses were performed on several ICP control methods. Our study provides a first attempt at making these comparisons and serves as a reference for intervention protocols.

## 2. Materials and Methods

### 2.1 Study Population

Patients who were managed at the PLA General Hospital between 2010 and 2023 and met the following criteria were recruited into the study: (1) had CSF opening pressure ≥250 mmH_2_O lasts for at least 14 days; (2) were aged 18 years or above; and (3) had complete clinical and follow-up data. All enrolled patients had received one or more medications to reduce ICP when raised CSF pressure was found for the first time. We divided the 119 cases into 5 groups, according to different treatment options used to manage ICP. Among them, 25 patients were treated with drug therapy alone (DT), 25 patients were treated with medications combined with repeated therapeutic LP, 23 were treated with medications combined with lumbar drainage (LD), 21 in combination with external ventricular drain (EVD) and 25 were treated with medications combined with an Ommaya reservoir (OR). The DT group served as the reference group and was compared with the other groups.

### 2.2 Clinical Data

The following clinical data were collected from each enrolled patient: age, gender, Body Mass Index (BMI) values, place of residence, past medical history, time from onset to the first visit, operation date, and indwelling time of several mechanical drainage measures, primary diseases, complications, sequelae, and outcomes. On average, patients underwent mechanical drainage procedures two weeks after being admitted. To ensure consistency in the observation time points, varying reference time points were established for each group based on the ICP-reducing treatment regimen they received. For non-surgical patients (DT and LP groups), the reference time point was defined as the week of admission. For patients who underwent mechanical drainage procedures, the reference time point was defined as the week of the procedure.

Baseline values were defined as those recorded before the reference time point, and efficacy was evaluated by comparing clinical data collected before and after this point. We recorded each patient’s Age-adjusted Charlson Comorbidity Index (ACCI), and modified Rankin Scale (mRS) scores, as well as the biochemical parameters prior to the reference time point and one and three months after the reference time point. In addition, we collected the CSF pressure values of all subjects before and after the reference time point at 1 week, 3 weeks, 6 weeks, 9 weeks, and 3 months from the medical consortium platform.

Patients were considered immunocompromised when they had the following conditions: malignancies, immune system diseases, nephrotic syndrome, active tuberculosis, cirrhosis, a history of corticosteroid use, and solid organ transplants. All enrolled patients received etiological and symptomatic treatment. Mannitol, glycerin fructose, hypertonic saline, human blood albumin, and diuretics were administered individually or in combination. At least 20 mL CSF was released during each operation of repeated therapeutic lumbar puncture [11]. Minimum follow-up time was 6 months. Patients whose condition deteriorated were followed up via telephone to determine the final outcome.

### 2.3 Statistical Analysis

Statistical analysis and graphical presentation of the data were performed in SPSS 23.0 (IBM Corporation, Armonk, NY, USA) and GraphPad Prism® 6 (GraphPad Software, Inc., San Diego, CA, USA) software. Continuous variables that conformed to normal distribution were presented as means ± standard deviation (SD), whereas those with skewed distributions were presented as medians (IQR = Q3–Q1). Differences among multiple groups were compared using one-way analysis of variance (ANOVA). When homogeneity of variance was met, Tukey’s test was performed for pairwise comparisons. When homogeneity of variance was not achieved, the Games-Howell method was used for multiple comparisons. If the condition of one-way ANOVA was not satisfied, we performed the nonparametric Kruskal-Wallis H test as appropriate. Binary-categorical variables in multi-group data were compared using the Chi-square test. In cases where the minimum expected count did not meet the requirement, we applied Fisher’s exact probability method. Repeated measures data were analyzed by generalized estimating equation (GEE) methods. We estimated main effects (group difference and time difference) and the interaction effect (time × group). When there was a significant interactive effect, one of the factors should be fixed for comparison. Pairwise comparison analysis was performed using the Least-Significant Difference test on GEE models. Statistical significance was set at *p* ≤ 0.05.

## 3. Results 

### 3.1 Baseline Demographics and Clinical Profiles

We found no statistically significant differences between the groups with regard to age, gender, BMI values, place of residence, immunodeficiency history, and constitution rate of primary diseases (Table 1). ACCI and mRS scores did not significantly differ between the intervention and control groups. Patients in the EVD group had shorter intervals from onset to first medical visit than their control counterparts, and no significant differences were observed between the remaining intervention groups and the DT group (Table 1).

**Table 1.** **Baseline characteristics of cases in each group**.

		DT	LP	LD	EVD	OR
Age, years, (mean ± SD)		48.00 ± 17.81	41.84 ± 18.83	43.85 ± 19.81	60.43 ± 15.28	47.36 ± 16.54
Sex, male, n (%)		17 (68.0)	12 (48.0)	14 (60.9)	12 (57.1)	12 (48.0)
BMI, kg/m^2^, (mean ± SD)		22.37 ± 4.17	23.15 ± 3.24	22.44 ± 3.10	24.40 ± 3.55	23.08 ± 3.97
Residence, city, n (%)		18 (72.0)	16 (64.0)	18 (78.3)	18 (85.7)	16 (64.0)
Immunodeficiency History, n (%)		9 (36.0)	7 (28.0)	8 (34.8)	5 (23.8)	11 (44.0)
Time intervals from onset to first medical visit, days, (mean ± SD)		34.36 ± 4.80	26.52 ± 3.67	21.70 ± 4.54	11.81 ± 3.51*	20.20 ± 3.70
ACCI score, median (IQR)		2 (4.0)	2 (2.5)	2 (3.0)	4 (3.0)	3 (2.5)
mRS score, median (IQR)		4 (2.0)	3 (1.5)	4 (1.0)	5 (1.5)	4 (1.5)
Primary diseases						
	CVD	6	4	7	12	4
	CNS infection	10	11	8	5	13
	Intracranial tumor	9	10	8	4	8

DT, drug therapy; LP, lumbar puncture; LD, lumbar drainage; EVD, external ventricular drain; OR, Ommaya reservoir; SD, standard deviation; n, number; BMI, Body Mass Index; ACCI, Age-adjusted Charlson Comorbidity Index; IQR, interquartile range; mRS, modified Rankin Scale; CVD, Cerebrovascular disease; CNS, central nervous system; Asterisks indicate statistical significance relative to control (*p < 0.05).

### *3.2* Changes in Liver and Kidney Function Indexes Before and After Intervention With Different ICP Reduction Measures

We fitted the GEE model to determine the trend in changes in the indexes of liver and renal functions across all groups. Grouping had no significant effect on the degree of changes in ALT and Tbil indicators. With the exception of creatinine (CRE), changes in the other clinical biochemical parameters were mainly driven by time (Table 2).

**Table 2.** **GEE results for the liver and kidney function indexes**.

	Group *p*	Time *p*	Group × time *p*
ALT	0.234	0.020*	0.077
AST	0.008**	0.000**	0.085
Tbil	0.858	0.005**	0.204
γ-GT	0.004**	0.000**	0.073
CRE	0.009**	0.690	0.148
BUN	0.010*	0.000**	0.112

ALT, alanine aminotransferase; AST, aspartate transaminase; Tbil, total bilirubin; γ-GT, γ-glutamyl transpeptidase; CRE, creatinine; BUN, blood urea nitrogen; Significance *p ≤ 0.05, **p < 0.01.

To further examine biochemical markers that displayed significant differences between groups, a comparative analysis was conducted. Specifically, the extent of change in the intervention group was compared to the control group while keeping time-point variables constant (Tables 3,4). Results showed that the median CRE value in the OR group significantly reduced at 3 months relative to the control group (Table 4). No significant difference was found in the other intervention groups.

**Table 3.** **Magnitude of changes in biochemical indicators at the 1 month across groups**.

	DT	LP	LD	EVD	OR
AST	1.11 (0.73)	1.10 (0.71)	1.31 (1.17)	1.74 (2.26)	1.25 (1.21)
γ-GT	1.17 (0.54)	1.18 (0.89)	0.72 (0.75)	1.67 (1.29)	1.23 (0.96)
CRE	1.09 (0.39)	1.00 (0.24)	0.94 (0.23)	0.89 (0.54)	0.86 (0.35)
BUN	1.12 (1.08)	1.07 (0.79)	1.08 (0.42)	1.66 (1.21)	0.93 (0.97)

Data were normalized to baselines values (% of corresponding baseline values). Data presented are medians (IQR). No significant difference was observed between intervention and control groups.

**Table 4.** **Magnitude of changes in biochemical indicators at the 3 months across groups**.

	DT	LP	LD	EVD	OR
AST	1.63 (1.16)	1.11 (0.88)	1.27 (1.32)	1.87 (2.17)	1.27 (1.44)
γ-GT	2.22 (2.05)	1.41 (1.33)	1.68 (2.77)	1.73 (1.34)	1.51 (1.34)
CRE	1.22 (0.44)	1.04 (0.27)	1.06 (0.44)	0.84 (0.49)	0.79 (0.49) *
BUN	1.41 (1.03)	1.10 (0.54)	1.17 (1.17)	1.66 (1.29)	0.87 (1.38)

*, Significantly different compared to control, *p* < 0.05.

### 3.3 mRS Scores

**Fig. 1.**
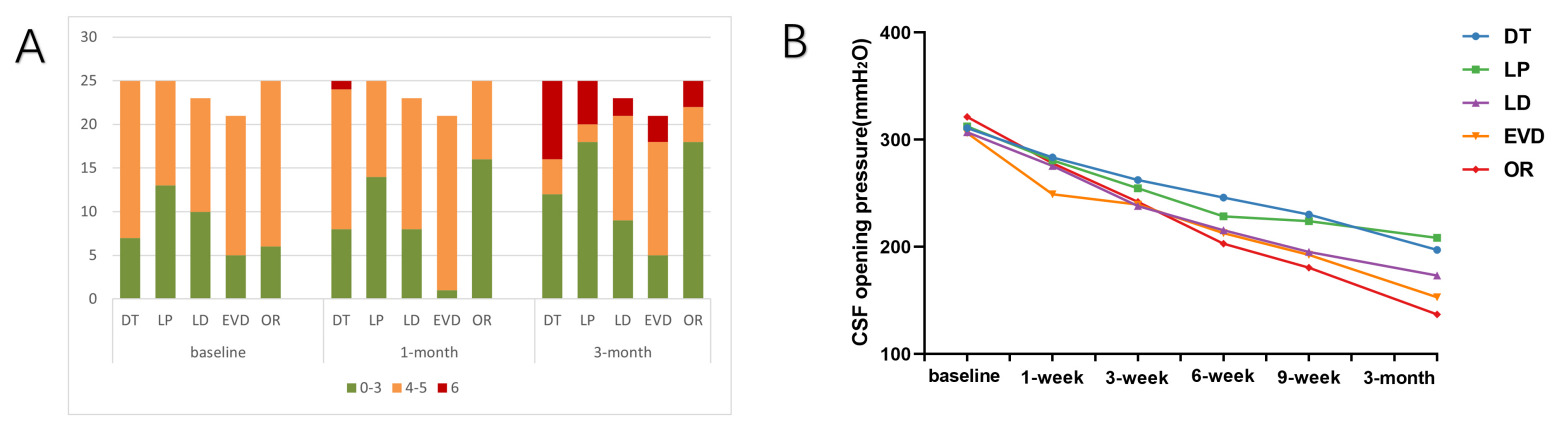
**Parameter values over time for each group**. (A) mRS scores recorded over time among the five groups. (B) Changes in intracranial pressure over time.

mRS scores of patients in each group were recorded at baseline, as well as 1 and 3 months after intervention (Fig. 1A). Summarily, between-group comparisons revealed statistically significant differences (Waldχ^2^ = 0.000 < 0.05). Estimated marginal means ± SD for each group were as follows: Patients in the OR, LP, EVD, LD and DT groups had means of 3.33 ± 0.20, 3.26 ± 0.22, 4.30 ± 0.13, 3.65 ± 0.18 and 3.95 ± 0.19, respectively. Scores were not significantly different between intervention and control groups at baseline, but were significant at 1 and 3 months in the OR (*p* = 0.003) and EVD (*p* = 0.018) groups respectively, relative to the control group. The LP group (*p* = 0.021) was also significantly different compared to the control group at 3 months. Notably, only patients who received Ommaya reservoirs demonstrated consistent improvement in their mRS scores compared to baseline.

Comparisons from repeated-measures (time) revealed statistically significant differences (Waldχ^2^ = 0.001 < 0.05). Marginal averages at baseline, 1 month, and 3 months were 3.92 ± 0.81, 3.60 ± 0.87 and 3.59 ± 0.13, respectively. Pairwise comparison revealed significantly different mRS scores in the OR group between baseline and all other time points after treatment (*p* = 0.000). In the LP group, mRS values at 1 month (*p* = 0.033) and 3 months (*p* = 0.026) were significantly different from these recorded at baseline. Moreover, we found significant interaction effects (group × time, Waldχ^2^ = 0.000 < 0.05), one of the factors should be fixed for pairwise comparison.

### 3.4 Comparison of ICP Across Groups

We employed GEE to analyze multiple groups of repeatedly measured intracranial pressure data and found statistically significant differences (Waldχ^2^ = 0.027 < 0.05). The OR, LP, EVD, LD, and DT groups had marginal means of 228.78 ± 8.40, 251.54 ± 9.62, 230.00 ± 7.42, 233.70 ± 8.0, and 256.83 ± 7.29, respectively. From week 6 post-treatment, the effect of reducing ICP was better in the OR group than that recorded in the other intervention groups (Fig. 1B). Repeated-measures (time) comparison results revealed statistically significant differences (Waldχ^2^ = 0.000 < 0.05).

The marginal averages at baseline and at 1, 3, 6, and 9 weeks were 311.59 ± 3.32, 273.38 ± 4.50, 247.65 ± 4.71, 222.97 ± 4.93, 205.54 ± 4.92, respectively, while 179.88 ± 4.85 was recorded at 3 months. Pairwise comparison revealed significant differences across all time points after treatment and baseline. Similarly, we observed significant interaction effects (group × time, Waldχ^2^ = 0.000 < 0.05), one of the factors should be fixed for pairwise comparison. There was a statistically significant difference between the OR and DT groups. This difference persisted and became more pronounced after 6 weeks from the overall trend.

### 3.5 Treatment Outcomes and Patient Prognosis

**Fig. 2.**
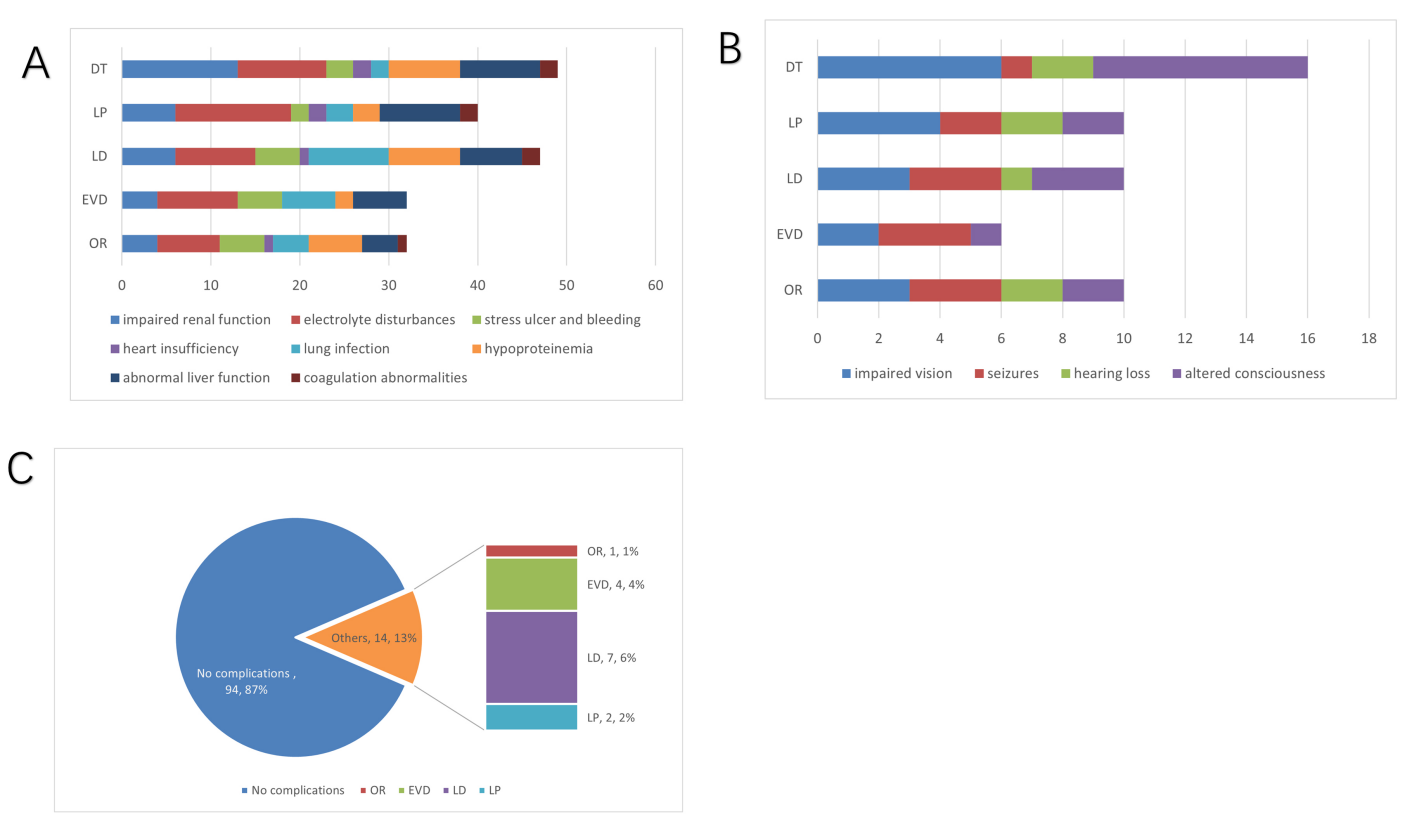
**Complication and sequelae**. (A) Major complications each group in comparison. If multiple complications occurred in the same patient, each was recorded each as a separate complication. (B) Profiles of sequelae incidence. (C) Incidence rates of surgical complications in each group.

Table 5 shows the fatality rates and incidence of sequelae for each group, with non-significant differences until the end of the follow-up period (Fig. 2A,B). Among them, patients in the OR group exhibited the lowest incidence of renal function deterioration and electrolyte disturbances, while those in the EVD and LD groups had a higher incidence of lung infection compared to the other intervention groups (Fig. 2A). Ninety-four enrolled patients had invasive procedures, with 14 patients experiencing surgical complications (Fig. 2C).

**Table 5.** **Case fatality ratio and sequelae rate at the end of follow up**.

	DT	LP	LD	EVD	OR
CFR	36% (9/25)	20% (5/25)	30.4% (7/23)	28.6% (6/21)	20% (5/25)
IOS	93.8% (15/16)	65% (13/20)	75% (12/16)	86.7% (13/15)	60% (12/20)

CFR, case fatality rate; IOS, incidence of sequelae. No statistically significant differences were observed among groups with regards to incidence of sequelae in surviving patients.

## 4. Discussion 

Persistent ICH is significantly associated with high patient mortality and poor prognosis [11, 12]. To prevent secondary injury and improve clinical outcomes, several clinical guidelines from various countries [13, 14, 15] have proposed the use of early and aggressive intervention. The neurocritical ill patients we focused on required management of ICP in months during treatment of primary disease. Aggressive treatment for reducing ICP can potentially improve the short-term prognosis more effectively than etiological treatment. Here, we sought to compare the efficacy of several frequently used regimens by retrospectively analyzing clinical data of patients with persistent ICH due to non-traumatic causes. We found that Ommaya reservoir has some advantages over other interventions.

Pharmacological treatments for persistent ICH include corticosteroids, acetazolamide, hypertonic saline, human albumin and mannitol, among others [15, 16]. In the initial stage, dehydration medications are widely used as part of first aid interventions. In the study, all enrolled patients were treated with personalized and active dehydration therapy. Drug therapy alone was not an appropriate option based on the data obtained. In practical clinical work, it is equally difficult to predict the duration of intracranial hypertension. However, prolonged dehydration treatment poses a considerable risk to renal and cardiac functions. Antibiotics and chemotherapy drugs that must be used in etiological treatment combined with dehydration drugs can increase the incidence of complications and sequelae.

The clinical guidelines of Infectious Diseases Society of America (IDSA) recommend repeated therapeutic LP to reduce ICH [11]. It is recommended that a sufficient volume of CSF (20–30 mL) be removed each time to reduce the initial opening pressure by up to 50%. If necessary, this operation can be performed daily. However, in the actual operation process, the frequency of lumbar puncture was inevitably affected by the subjective will of patients. The longer the treatment, the more difficult it is for patients to tolerate. In our study, no obvious advantage of the LP group over the DT group was observed, which could be attributed to this. There is a risk of wound infection and brain herniation during the procedure of frequent lumbar puncture that demands special attention. Lumbar puncture did not affect the patients’ neurological rehabilitation training, which was beneficial for improving the mRS score.

LD and EVD are similar mechanical drainage patterns that can continuously drain and control the flow rate [17, 18]. The operation is relatively simple and effective, and is widely used in clinics. However, some shortcomings can be observed in the study. Drainage tubes must be replaced regularly. The lumbar cistern drainage tube needs to be replaced within 7–10 days, while the external ventricular drain tube needs to be replaced within about 14 days. Patients with CNS infections and tumor diseases had elevated CSF protein levels, resulting in catheter jams and premature replacement. One end of the drainage tube is always exposed, which increases the difficulty of nursing [19]. In reality, patients carrying drainage devices are severely restricted in their posture and range of motion. This not only prolongs the time in bed but also increases the risk of complications. Compared to the DT group, the LD and EVD groups showed no statistically significant differences in mRS scores, with higher rates of lung infection.

The Ommaya reservoir, a type of ventricular drainage system invented by Ommaya in 1963, comprises a flat reservoir buried in the periosteum and a drainage catheter that is inserted into the lateral ventricle’s anterior horn [20]. Currently, the Ommaya reservoir is widely used for intermittent intraventricular administration of chemotherapeutic drugs [21]. However, its efficacy in controlling ICP has not yet been compared to other measures. After the implantation of the Ommaya reservoir, CSF is drained through a minimally invasive and closed approach. The device not only avoids the pain caused by repeated lumbar punctures, but it also relieves the posture restrictions caused by wearing drainage devices. When the drainage tube is clamped, the patient can freely carry the reservoir, thus greatly improving comfort during the treatment process. The patient’s willingness to retain a drainage device for a long time is a prerequisite for effective control of ICP. Meanwhile, the relatively closed drainage device can effectively reduce the risk of infection, especially when patients returned to the medical consortium near their homes to complete subsequent treatment. In this study, Ommaya reservoir effectively reduced ICP, as evidenced by a stable and sustained effect starting from week 6 post-placement. Well-controlled ICP mediated a reduction in mRS scores thereby improving patient status in the OR group (Fig. 1A). The device can also reduce the risk of electrolyte disorders, hypoproteinemia, and stress ulcers (Fig. 2A). The intervention of the Ommaya reservoir resulted in a decrease in CRE value at 3 months, which was associated with reduced use of dehydration drugs. GEE results revealed no significant group-by-time interaction effect. It is suggested that renal function was mainly affected by intervention measures rather than being time-dependent.

The complications associated with Ommaya placement include intracranial hematoma, seizure and second infection, among others [22]. The incidence of intracranial hemorrhage is 1.3% and infection incidence ranges from 3 to 15% [23, 24]. Previous studies suggested that there was no correlation between the number of times the reservoir was punctured and the chance of infection [25, 26]. The occurrence of infection may be related to inadequate techniques of skin preparation and reservoir entry [27]. Numerous clinical experiences have shown that complications associated with Ommaya reservoir placement are controllable [22, 28]. The longest indwelling time of Ommaya reservoir in the study was 608 days. Of these, only one patient changed the device early due to poor drainage (Fig. 2C). Removal of the Ommaya reservoir is a conventional and comparatively safe surgical procedure, and its risk is usually much lower than that of initial placement. The main operation is placed on the scalp and skull surface and does not involve intraventricular puncture. The risk of infection and anesthesia exists in all surgical procedures. To conclude, it is extremely rare that removal of the Ommaya reservoir leads to neurological impairment.

This retrospective study has some issues that require additional explanation. First of all, the selection bias of medical decision-making in the study was inevitable. For patients in good general condition at the initial stage, the physician may prefer the scheme without implants (DT or LP groups) for controlling ICH. Furthermore, consent from patients and their families must be obtained for surgical procedures, and their wishes can influence treatment choices. Secondly, although there was no statistical difference in the disease ratios among the groups, the number of patients with cerebrovascular disease in the EVD group was larger. Cerebrovascular disease develops rapidly, prompting patients to seek medical attention sooner. As a result, the time between symptom onset and the initial medical consultation in the EVD group was slightly shorter than in the other groups. Third, we believe that short-term case-fatality rates were associated with ICP reduction effects, while long-term case-fatality rates were linked to the primary disease. For incurable diseases, controlling ICP can only delay progression, not change final outcomes. Finally, we could not develop an effective prediction model for in-depth correlation analysis due to the limited number of cases in the study. The establishment of the medical consortium ensures sequential treatment and data collection. We will gather more data from the platform and develop an effective model in the future.

## 5. Conclusions

In summary, Ommaya reservoir is a safe and effective device for controlling ICP. It can prevent brain herniation, protect renal function, improve patients’ general state and outcomes. This device is beneficial for management of early stages of persistent ICH.

## Availability of Data and Materials

The sets of data generated and analyzed during the course of the study cannot be shared publicly due to legal restrictions, but are available from the corresponding author upon reasonable request.
